# Identifying Predictors of Pathological Complete Response in Moroccan Patients With HER2-Positive Breast Cancer Treated With Neoadjuvant Therapy

**DOI:** 10.7759/cureus.112103

**Published:** 2026-07-05

**Authors:** Boutaina Atmane, Jihad El Afeya, Safae Toumi, Hanane Inrhaoun, Rachid Razine, Hind Mrabti, Hassan Errihani

**Affiliations:** 1 Department of Medical Oncology, Institut National d'Oncologie, Mohammed V University, Rabat, MAR; 2 Department of Biostatistics and Epidemiology, Faculty of Medicine and Pharmacy of Rabat, Mohammed V University, Rabat, MAR

**Keywords:** breast cancer, her2, morocco, pathological complete response, prognostic factors

## Abstract

Background

The prognosis of human epidermal growth factor receptor 2 (HER2)-positive breast cancer has significantly improved with the use of neoadjuvant chemotherapy combined with anti-HER2 therapy. Achieving pathological complete response (pCR) is commonly associated with favorable long-term outcomes. However, real-world data on predictive factors influencing pCR remain limited, particularly in Morocco and North Africa, where evidence is scarce. Generating region-specific evidence is therefore essential to complement findings from other populations. This study aimed to assess pCR rates and identify predictors of response in Moroccan patients with HER2-positive breast cancer treated with neoadjuvant systemic therapy.

Methods

We retrospectively included 203 patients with stage I-III HER2-positive breast cancer treated with neoadjuvant chemotherapy plus trastuzumab, with or without pertuzumab, followed by surgery between October 2019 and April 2023. Predictors of pCR were assessed using logistic regression. Disease-free survival (DFS) was compared between patients achieving pCR and those without pCR using Cox proportional hazards regression analysis.

Results

Among 203 patients, 44.3% achieved pCR. Higher pCR rates were independently associated with hormone receptor-negative tumors (odds ratio (OR) = 2.20, 95% confidence interval (CI): 1.08-4.47), HER2 (score 3) status (OR = 4.80, 95% CI: 1.73-13.30), and the use of dual HER2 blockade (OR = 2.05, 95% CI: 1.03-4.07) compared with triple-positive tumors and HER2 (score 2) fluorescence in situ hybridization (FISH)-amplified tumors treated with trastuzumab alone. In contrast, lower pCR rates were observed in T3-T4 tumors (OR = 0.45, 95% CI: 0.23-0.92). After a median follow-up of 51.3 months, achieving pCR was significantly associated with improved DFS (HR = 0.07, 95% CI: 0.02-0.28; p < 0.001).

Conclusions

Tumor stage, hormone receptor status, HER2 expression, and neoadjuvant pertuzumab use were independently associated with pCR, emphasizing the importance of tailored neoadjuvant strategies in HER2-positive breast cancer.

## Introduction

Breast cancer is the most frequently diagnosed cancer among women worldwide [1]. In Morocco, it accounts for 39.1% of all female cancers, representing a major public health burden [2]. Approximately 20% of breast cancers are characterized by overexpression or amplification of the human epidermal growth factor receptor 2 (HER2), defining a biologically aggressive subtype historically associated with poor prognosis [3]. HER2 overexpression was observed in approximately 29% of Moroccan women with breast cancer [4,5]. The introduction of HER2-targeted therapies has markedly improved the prognosis of these patients. Current standard management of HER2-positive early breast cancer includes neoadjuvant chemotherapy combined with anti-HER2 agents [6]. This therapeutic approach not only increases the rate of breast-conserving surgery but also allows for treatment individualization based on the pathological response to neoadjuvant therapy. Pathological complete response (pCR), defined as the absence of residual invasive disease in both the breast and regional lymph nodes following neoadjuvant systemic treatment, is considered a robust surrogate marker reflecting improved long-term clinical outcomes [7-9].

Multiple clinical trials and real-world studies have consistently demonstrated that patients achieving pCR after neoadjuvant therapy experience significantly improved prognoses compared with those with residual disease [10]. Nonetheless, HER2-positive breast cancer remains a biologically heterogeneous disease, and response to neoadjuvant therapy varies considerably among patients [11]. The factors predicting pCR are not yet fully elucidated, particularly in real-world settings and across different populations [12].

In Morocco, data regarding treatment response and predictive factors of pCR in HER2-positive breast cancer are limited. Given potential differences in tumor characteristics, access to therapies, and patient demographics, it is essential to evaluate real-world outcomes within this population. This study aimed to identify predictors of pCR in Moroccan patients with HER2-positive breast cancer receiving neoadjuvant chemotherapy plus anti-HER2 therapy, and to evaluate the association between pCR and disease-free survival (DFS).

Preliminary findings from this study were presented as a poster at the European Society for Clinical Oncology (ESMO) Breast Cancer Annual Congress held on May 6, 2026.

## Materials and methods

Study population

We conducted a retrospective cohort study of patients with HER2-positive invasive breast cancer diagnosed between October 2019 and April 2023 at the National Institute of Oncology in Rabat, who received neoadjuvant therapy followed by surgery. Eligible patients had newly diagnosed stage I-III disease confirmed by core needle biopsy, HER2-positive status, and were treated with neoadjuvant chemotherapy in combination with trastuzumab, with or without pertuzumab, followed by curative-intent surgery and postoperative pathological assessment.

Patients were excluded if they were male, were pregnant during treatment, presented with metastatic disease at diagnosis, had synchronous bilateral breast cancer, did not receive neoadjuvant therapy, experienced disease progression during neoadjuvant treatment, did not undergo surgery, or had incomplete clinicopathological data. Among the patients who did not receive neoadjuvant therapy, 10 had T1N0 disease treated with upfront surgery according to standard management of early-stage breast cancer, while the remaining patients were referred postoperatively for adjuvant systemic treatment and therefore did not enter the neoadjuvant treatment pathway evaluated in this study.

Study variables

Data collected included age at diagnosis, menopausal status, body mass index (BMI), clinical tumor and nodal stage, hormone receptor (HR) status, HER2 immunohistochemical (IHC) score, Ki-67 index, HER2-targeted regimens, surgery delay, and postoperative pathological findings. All patients received a standardized anthracycline- and taxane-based neoadjuvant chemotherapy backbone, consisting of either epirubicin/cyclophosphamide (EC) or doxorubicin/cyclophosphamide (AC), followed by docetaxel or weekly paclitaxel.

Clinical nodal status (cN) was assessed at baseline based on physical examination and imaging. Fine-needle aspiration or core biopsy of lymph nodes was not performed systematically and was reserved for selected cases.

HER2 and HR expression were assessed according to American Society of Clinical Oncology/College of American Pathologists guidelines [13,14]. HER2 positivity was defined as an IHC score of 3+ or 2+ with confirmed amplification by fluorescence in situ hybridization (FISH). Tumors were considered estrogen receptor (ER)- or progesterone receptor (PR)-positive if ≥1% of tumor cell nuclei stained positive by IHC. According to ER and PR expression profiles, patients were grouped into three categories: triple-positive (triple-positive breast cancer (TPBC): ER+/PR+/HER2+), single HR+/HER2+ (ER+/PR−/HER2+ or ER−/PR+/HER2+), and HR-/HER2+ (ER−/PR−/HER2+).

Patients were classified according to neoadjuvant HER2-targeted therapy as receiving either trastuzumab plus pertuzumab (T + P) or trastuzumab alone (T). The use of pertuzumab depended on its availability in the hospital pharmacy, as temporary stock shortages occurred during the study period. Surgical delay was defined as the interval (in days) between completion of neoadjuvant chemotherapy combined with HER2-targeted therapy and surgery. An eight-week cut-off was used to categorize surgical delay (<8 weeks vs. ≥8 weeks), based on previously reported clinically relevant thresholds in the literature [15]. DFS was defined as the time from the initial diagnosis to the recurrence of the disease.

Sample size

Our sample size was determined to ensure adequate statistical power to detect clinically meaningful associations between candidate predictors and pCR in HER2-positive breast cancer treated with neoadjuvant chemotherapy plus anti-HER2 therapy. Based on contemporary cohorts, pCR rates in this setting range from approximately 45% to 70% [16-18]. Assuming a conservative expected pCR rate of 50%, a two-sided type I error α = 0.05, and power (1 − β) = 80% (β = 0.20), and applying the rule of at least 10 events per predictor variable for multivariable logistic regression, a minimum of 200 patients is required to reliably evaluate about 10 independent covariates (≈100 pCR events and 100 non-pCR events).

Statistical analysis

Statistical analyses were performed using Jamovi (version 2.6.2). Quantitative variables are presented as means ± standard deviation or medians with interquartile ranges (IQRs); categorical variables are reported as frequencies and percentages. Variables with more than 25% missing data were excluded from the analyses. No imputation of missing values was performed, and analyses were conducted using available data only. Ki-67 was not routinely assessed in HER2-positive breast cancer in our institution, resulting in substantial missing data (89 available, 114 missing), and was therefore excluded from the final analyses.

Associations between clinicopathological variables and pCR were first assessed using logistic regression analysis. Variables with p < 0.1 in univariable analysis, along with clinically relevant factors, were included in multivariable logistic regression to identify independent predictors of pCR. Results are reported as odds ratios (ORs) with 95% confidence intervals (CIs). A two-sided p-value < 0.05 was considered statistically significant. DFS was evaluated as a secondary outcome using Cox proportional hazards regression. Survival curves were estimated using the Kaplan-Meier method and compared according to pCR status using the log-rank test.

## Results

Clinicopathologic features of patients

**Figure 1:**
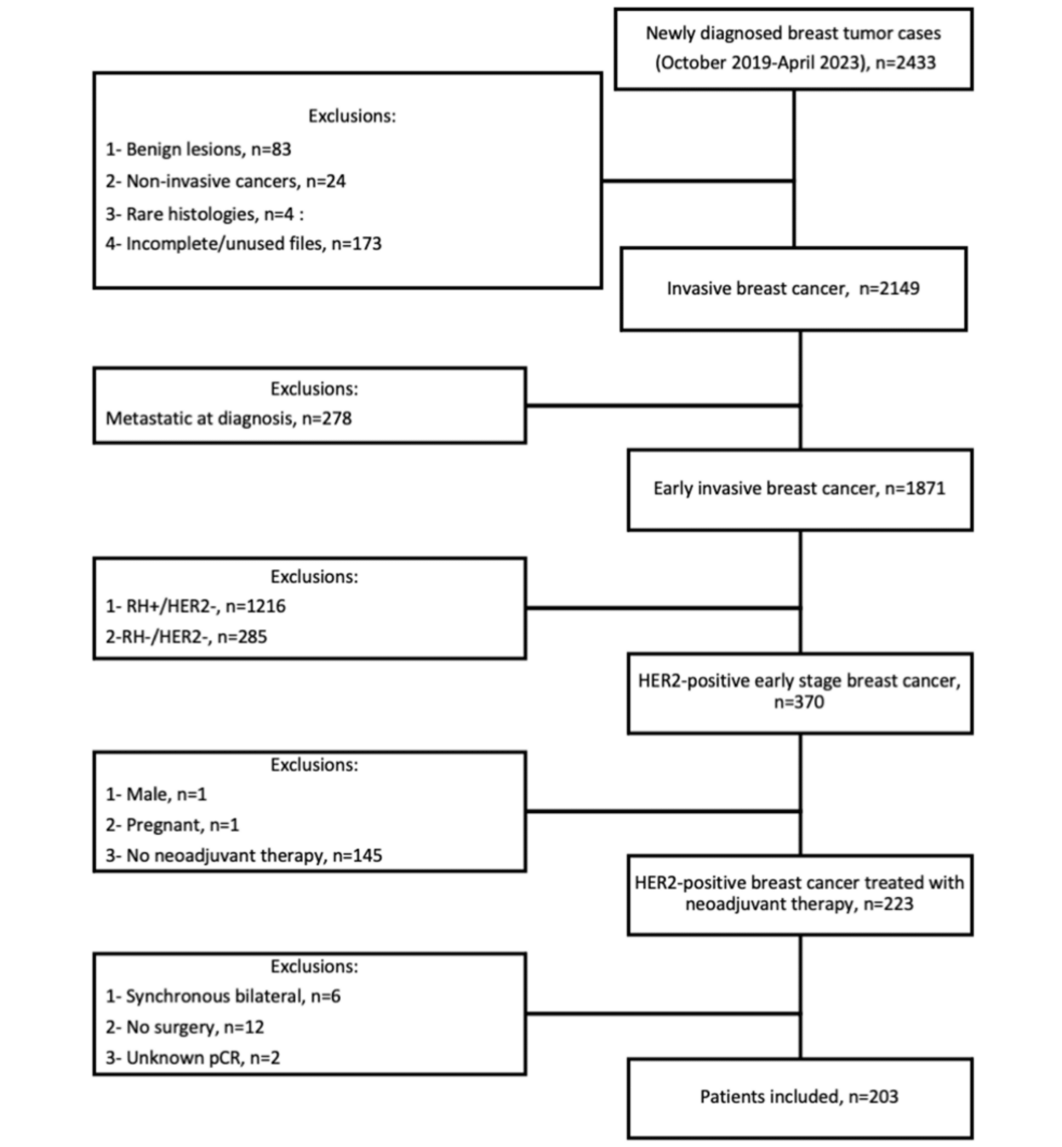
Patient selection flowchart pCR: pathological complete response; HER2: human epidermal growth factor receptor 2

Among 1,871 patients diagnosed with early breast cancer from October 2019 to April 2023, 370 (19.77%) were HER2-positive. Of these, 203 met the inclusion criteria for the present study. The remaining patients were excluded mainly due to upfront surgery followed by adjuvant treatment or failure to meet inclusion criteria (Figure 1).

The median age at diagnosis was 49 (43, 58) years, with 40 (19.7%) patients aged under 40 years. At presentation, 124 (61.6%) had tumors classified as T2. HER2 (score 3) disease was observed in 171 (84.2%) patients. TPBC was observed in 96 (47.3%) cases. All patients received anthracycline- and taxane-based chemotherapy in combination with HER2-targeted therapy. Dual HER2 blockade was administered in 76 (37.4%) patients. Following neoadjuvant therapy, 90 (44.3%) patients achieved a pCR (Table 1).

**Table 1:** Patient characteristics ^a^Expressed as medians and interquartile ranges ^b^Expressed as counts and percentages BMI: body mass index; HER2: human epidermal growth factor 2; HR: hormone receptor; TPBC: triple-positive breast cancer; cT: clinical tumor stage; cN: clinical lymph node stage; T: trastuzumab; P: pertuzumab; pCR: pathological complete response

Clinicopathological parameters	Value
Age (years)^a^	49.0 (43.0, 58.0)
BMI^b^	
≤25	70 (34.5)
>25	133 (65.5)
HER2 status^b^	
Score 2	32 (15.8)
Score 3	171 (84.2)
HR status^b^	
TPBC	96 (47.3)
Single HR+/HER2+	34 (16.7)
HR-/HER2+	73 (36.0)
Ki-67 index%^a^	35.0 (22.0, 48.0)
Grade^b^	
I-II	117 (57.6)
III	86 (42.4)
cT stage^b^	
cT1-cT2	135 (66.5)
cT3-cT4	68 (33.5)
cN stage^b^	
cN0	70 (34.5)
cN+	133 (65.5)
HER2-targeted therapy^b^	
T	127 (62.6)
T + P	76 (37.4)
Surgery delay (days)^b^	
≤8 weeks	126 (64.0)
>8 weeks	71 (36.0)
Treatment outcome^b^	
pCR	90 (44.3)
Relapse	34 (16.7)

Predictive factors of pCR

Predictive factors associated with pCR are summarized in Table 2. In multivariate analysis, HR-negative status, HER2 (score 3+) expression, and dual HER2 blockade emerged as independent predictors of pCR, whereas larger tumor size was independently associated with a lower likelihood of achieving pCR.

**Table 2:** Predictive factors of pCR in univariate and multivariate analyses R: reference category; OR: odds ratio; CI: confidence interval; BMI: body mass index; TPBC: triple-positive breast cancer; cT: clinical tumor stage; cN: clinical lymph node stage; HER2: human epidermal growth factor 2; HR: hormone receptor; T: trastuzumab; P: pertuzumab; pCR: pathological complete response

Variable	Univariate analysis		Multivariate analysis	
	OR (95% CI)	p-value	OR (95% CI)	p-value
Age				
≤40 years (R)	1		1	
>40 years	1.86 (0.89-3.85)	0.096	1.33 (0.59-3.02)	0.484
BMI				
≤25 (R)	1		-	-
>25	0.70 (0.39-1.26)	0.239	-	-
Grade				
I-II (R)	1		1	
III	1.75 (0.99-3.08)	0.050	1.66 (0.89-3.13)	0.114
cT stage				
T1-T2 (R)	1		1	
T3-T4	0.20 (0.24-0.72)	0.003	0.45 (0.23-0.92)	0.027
cN stage				
N0 (R)	1		1	
N+	0.49 (0.27-0.89)	0.019	0.56 (0.28-1.09)	0.089
HER2 status				
Score 2 (R)	1		1	
Score 3	3.36 (1.38-8.20)	0.007	4.80 (1.73-13.30)	0.002
HR status				
TPBC (R)	1		1	
Single HR+/HER2+	1.82 (0.82-4.02)	0.137	2.06 (0.82-5.16)	0.123
HR-/HER2+	2.09 (1.12-3.89)	0.020	2.20 (1.08-4.47)	0.029
Anti-HER2				
T (R)	1		1	
T + P	1.57 (0.88-2.78)	0.122	2.05 (1.03-4.07)	0.039
Surgery delay				
≤8 weeks (R)	1		1	
>8 weeks	0.59 (0.32-1.08)	0.089	0.51 (0.26-1.01)	0.055

Survival analysis 

**Figure 2:**
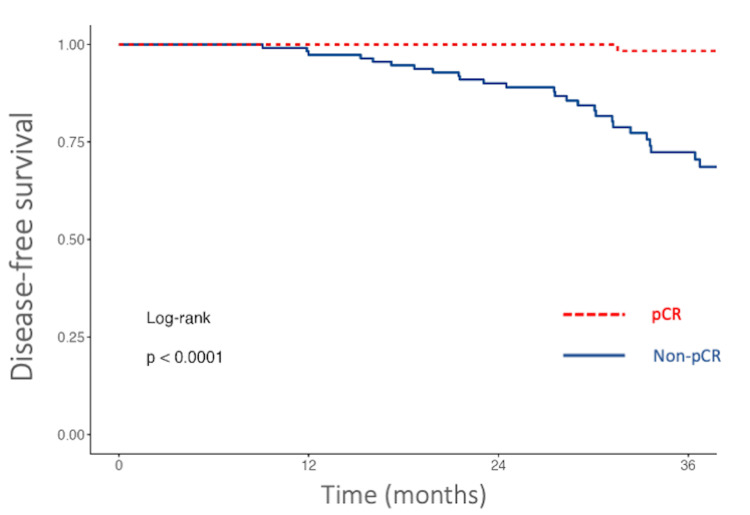
Estimated DFS analysis according to pCR DFS: disease-free survival; pCR: pathological complete response

The median follow-up was 51.3 months (48.4-52.2), during which 34 patients experienced relapse. Achievement of pCR was associated with improved DFS (HR = 0.07, 95% CI: 0.02-0.28; p < 0.001). The three-year DFS rates were 98.3% in patients with pCR and 72.3% in those without pCR (Figure 2).

## Discussion

In this study, higher pCR rates were observed in patients with HR-negative tumors, HER2 overexpression (IHC 3+), and those receiving dual HER2 blockade with pertuzumab. Conversely, lower pCR rates were associated with advanced tumor stage (T3-T4). The overall pCR rate in our cohort was 44.3%, comparable to that reported in the NOAH trial (38%) with trastuzumab-based neoadjuvant chemotherapy and the NeoSphere trial (45.8%) evaluating dual HER2 blockade [19,20]. These results are consistent with another Moroccan real-world study in the same setting, which reported a pCR rate of 51.4% [21]. Overall, both studies suggest similar real-world outcomes in Moroccan patients treated with neoadjuvant anti-HER2 therapy, with variability likely reflecting differences in baseline tumor characteristics and disease.

Higher pCR rates (>60%) have been reported in the more recent TRYPHAENA and TRAIN-2 trials [22,23]. The higher pCR rates observed in these studies may be explained by differences in treatment protocols. In both trials, dual HER2 blockade with trastuzumab and pertuzumab was routinely administered according to standardized protocols, whereas only 37.4% of patients in our cohort received pertuzumab. Moreover, unlike our cohort, chemotherapy regimens in the TRAIN-2 trial incorporated carboplatin in combination with dual HER2 blockade, whereas all patients in our cohort received conventional anthracycline- and taxane-based chemotherapy. These differences in treatment strategies and access to anti-HER2 therapy likely contributed to the lower pCR rate observed in our real-world cohort. Nevertheless, consistent with evidence from randomized trials, dual HER2 blockade remained an independent predictor of pCR in our study (OR = 2.05, 95% CI: 1.03-4.07; p = 0.039), underscoring the importance of improving access to pertuzumab in resource-limited settings.

Several studies have shown that patients with HER2 (score 3) tumors achieve pCR significantly more frequently than those with HER2 (score 2) with FISH amplification, a finding that our study confirms, highlighting the greater benefit of neoadjuvant therapy in tumors with strong HER2 overexpression [24,25].

Higher pCR rates were also observed in HR-negative tumors compared with TPBC. Similarly, Chen et al. demonstrated that TPBC is an independent predictor of poor response to neoadjuvant therapy [26]. This may be partly explained by the biological interplay between the HER2 and HR pathways. In HR+/HE2+ breast cancer, inhibition of HER2 signaling can lead to reactivation of HR signaling, a well-recognized mechanism of resistance to anti-HER2 therapies [27].

Regarding surgical delays, a recent meta-analysis reported that patients undergoing surgery more than eight weeks after neoadjuvant chemotherapy had significantly worse overall survival. However, no differences in DFS or pCR rates were observed when comparing surgery delays [28]. In our cohort, we found no statistically significant association between surgical delay and pCR in multivariable analysis (OR = 0.51, 95% CI: 0.26-1.01; p = 0.055), although a non-significant trend was observed, suggesting limited clinical relevance.

Accumulating evidence indicates that achieving pCR is strongly associated with long-term clinical benefit in HER2-positive breast cancer [9]. In our cohort, achieving pCR was strongly associated with improved DFS (HR = 0.07, p < 0.001), confirming its role as a robust prognostic biomarker in HER2-positive breast cancer.

This study represents one of the largest single-center real-world cohorts evaluating response to neoadjuvant chemotherapy combined with anti-HER2 therapy in Moroccan patients with early or locally advanced HER2-positive breast cancer. To our knowledge, previous Moroccan real-world evidence in this setting remains limited, with the largest published series including 140 patients [21], highlighting the relatively substantial size of our cohort and its contribution to the local evidence base. Our findings provide additional insights into clinicopathological factors associated with pCR in routine clinical practice. Despite these strengths, several limitations should be acknowledged. The retrospective, single-center design inherently limits causal inference and may introduce selection bias and residual confounding that could not be fully controlled. Although the overall sample size was sufficient for the main analyses, some predefined subgroups included relatively small numbers of patients, which may limit the robustness of subgroup comparisons. In addition, missing data for Ki-67, despite its recognized prognostic and predictive value, prevented its inclusion in multivariable analyses. Surgical delay variables were not systematically captured, precluding a detailed assessment of potential contributing factors, including the impact of the COVID-19 pandemic. Finally, the relatively short follow-up period limits the evaluation of long-term outcomes, particularly disease-free and overall survival.

## Conclusions

In this study, we evaluated pCR rates and identified predictive factors of response in a Moroccan cohort of patients with HER2-positive breast cancer treated with neoadjuvant systemic therapy. We observed that tumor stage, HR status, HER2 expression level, and the use of neoadjuvant pertuzumab were independent predictors of pCR. These findings reinforce the heterogeneity of HER2-positive breast cancer and the variability in response to standard neoadjuvant regimens in real-world settings. Our results may support a more individualized therapeutic approach based on baseline tumor characteristics and treatment selection to optimize the likelihood of achieving pCR. Finally, prospective multicenter studies are warranted to validate these results and improve the generalizability of evidence across the region.
